# ‘Messy’ Processing of χ-conotoxin MrIA Generates Homologues with Reduced hNET Potency

**DOI:** 10.3390/md17030165

**Published:** 2019-03-14

**Authors:** Rebekah Ziegman, Andreas Brust, Prerna Jha, Fernanda C. Cardoso, Richard J. Lewis, Paul F. Alewood

**Affiliations:** Institute for Molecular Bioscience, The University of Queensland, St. Lucia, Queensland 4072, Australia; r.ziegman@uq.edu.au (R.Z.); a.brust@imb.uq.edu.au (A.B.); p.jha@imb.uq.edu.au (P.J.); f.caldascardoso@uq.edu.au (F.C.C.); r.lewis@uq.edu.au (R.J.L.)

**Keywords:** conotoxin, MrIA, peptides, Norepinephrine Transporter Inhibitor (NET), bioactivity

## Abstract

Integrated venomics techniques have shown that variable processing of conotoxins from *Conus marmoreus* resulted in a dramatic expansion in the number of expressed conotoxins. One conotoxin from *C. marmoreus*, the χ-conotoxin MrIA, is a selective inhibitor of human norepinephrine transporters (hNET) and therefore a drug candidate for attenuating chronic neuropathic pain. It has been found that “messy” processing of the MrIA transcripts results in the expression of MrIA analogs with different truncations of the pro-peptide that contains portions of the MrIA molecule. The aim of this study was to investigate if variable processing of the expressed peptides results in modulation of the existing hNET pharmacology or creates new pharmacologies. To this end, a number of MrIA analogs found in *C. marmoreus* venom were synthesized and evaluated for their activity at hNET receptors. While several of the analogs exhibited norepinephrine transporter inhibitory activity comparable to that of MrIA, none significantly improved on the potency of conotoxin MrIA, and those analogs with disrupted pharmacophores produced greatly reduced NET inhibition, confirming previous structure-activity relationships seen on χ-class conopeptides. Additionally, analogs were screened for new activities on ion channels using calcium influx assays, although no major new pharmacology was revealed.

## 1. Introduction

Cone snails represent a large taxon of carnivorous gastropods that use specialized venom to hunt fish, worms, or fellow molluscs. The venom of these snails is prolific with small, disulfide-rich peptides that bind to various physiological targets. Because the disulfide bonding leads to highly stable and constrained 3-dimensional conformations [1], conotoxins, as they are known, have repeatedly shown high specificity for their targets, making them an excellent resource for the discovery of novel, medically significant compounds [1,2,3].

**Figure 1:**
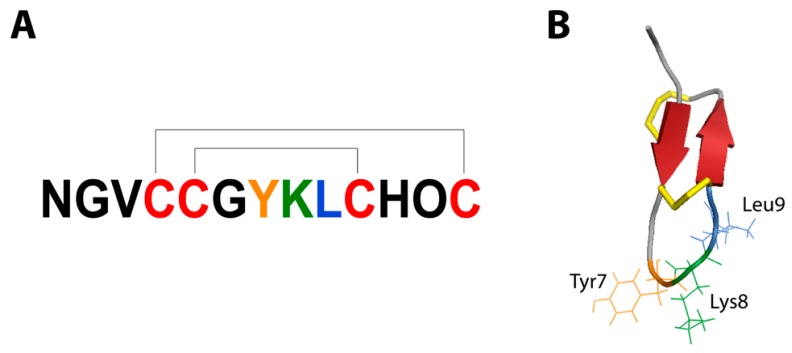
(**A**) Primary structure of MrIA showing Cys1-Cys3, Cys2-Cys4 disulfide connectivity, Cys residues are coloured red, (**B**) 3D structure of MrIA. Yellow portions represent disulfide bonds and red arrows represent β-sheets. Pharmacophore residues are coloured orange (Tyr), green (Lys), and blue (Leu).

Due to recent transcriptomic sequencing of *Conus* venom glands, the discovery rate of novel conotoxins has increased dramatically [4,5,6]. Additionally, it has been revealed that, despite the large number of conotoxins found in a single snail’s venom, they originate from a relatively small number of genes [4]. In fact, it has been found that in a single species of cone snail, approximately only 100 genes are responsible for producing thousands of peptides [4]. This molecular diversity is possible via variable peptide processing (VPP), in which the use of alternative cleavage sites, post-translational modifications (PTMs), and variable N- and C-terminal truncations create a plethora of peptides from a single gene precursor, resulting in biological “messiness” at the proteomic level. Of particular interest was the gene coding for the χ-conotoxin MrIA (sequence NGVCCGYKLCHOC-NH2) because of its proven pharmacological relevance and its high expression in the venom. MrIA specifically inhibits human norepinephrine transporters (hNET) at an allosteric site, leading to an attenuation of neuropathic pain [7]. Because of this, an optimized version of MrIA, known as Xen2174 (sequence: ZGVCCGYKLCHOC-NH_2_), was progressed into phase II clinical trials to treat pain in post-surgical and cancer patients [8]. The high hNET selectivity of MrIA’s targeting is modulated by its pharmacophore, which is well understood [9]. The pharmacophore includes the stabilizing scaffold of two disulfide bonds joined in a 1-4, 2-3 ribbon connectivity. The scaffold stabilizes the pharmacophore residues, Tyr7, Lys8, and Leu9, and creates an inverse gamma turn that presents the pharmacophore residues and allows for selective binding on the hNET target [9] (Figure 1). It was found that modifications to any of the pharmacophore residues as well as slight structural changes could have large impacts on the hNET inhibition exhibited by the peptide [9].

In the study by Dutertre et al. [4] on *C. marmoreus* venom, 72 unique peptide masses related to MrIA were identified via proteomic methods that corresponded to various peptides originating from the MrIA parent peptide. A variety of different truncations contributed to this remarkable diversity, as well as PTMs, including C-terminal amidation and the inclusion of non-typical amino acids, such as pyroglutamic acid. MrIA and its deamidated form were much more dominant in the venom, with the next most intense mass precursor ion having an intensity of only approximately 4% the intensity of the deamidated form and 90% of the peptides with intensities of less than 1% of MrIA [4]. Currently, it is unknown how, if at all, peptides expressed at such low levels affect venom lethality.

A number of the MrIA analogs identified contained either the entire MrIA pharmacophore [4], or portions of it. However, the purpose of the analogs as venom components is unclear. Therefore, the aim of the present study was to investigate the activity of these MrIA analogs on hNET and ion channels that act as common conotoxin targets to gain a better understanding of their biological significance.

## 2. Results and Discussion

### 2.1. Peptide Synthesis

From the 72 MrIA analogs identified by Dutertre et al. [4], 18 sequences were chosen to be synthesized based on several factors, including size, the modified residues present, and the inclusion of fragments of the MrIA pharmacophore. Following synthesis and oxidation, a total of 26 peptide analogues were obtained, including O12P-MrIA in both its amidated **1** and acidic forms **2** (Table 1, **1** and **2**, respectively). The total number of analogs obtained exceeded the number of sequences used, as several sequences produced disulfide isomers upon oxidation (Table 1). Additionally, those analogs containing three cysteine residues produced dimers when oxidized.

### 2.2. Norepinephrine Uptake Inhibition Assay

**Figure 2:**
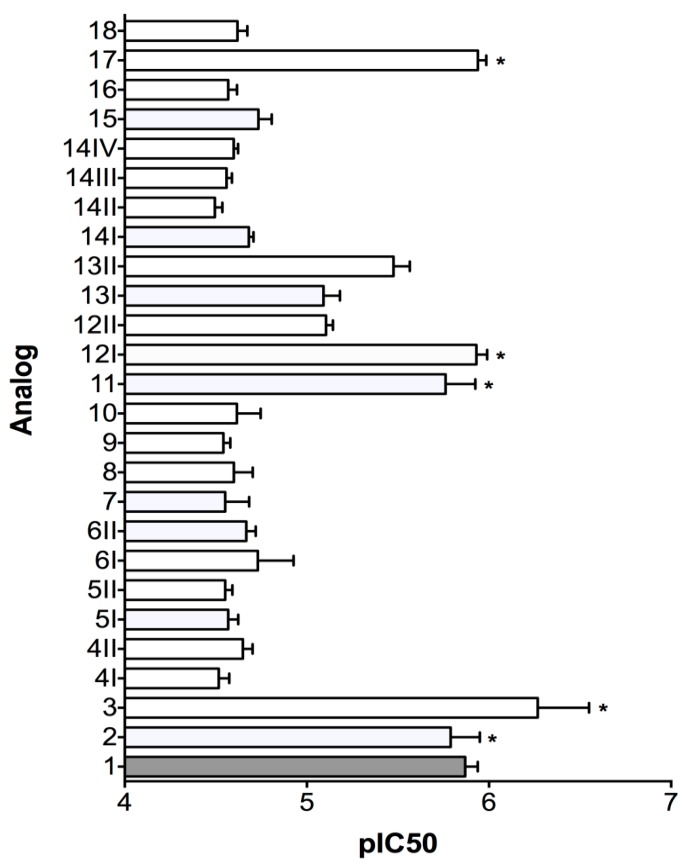
pIC_50_ values of MrIA analogs against human norepinephrine transporter (hNET) and their standard deviation bars. The MrIA standard (**1**) is coloured grey. Analogs whose hNET inhibitory activity do not significantly differ from the standard are marked by *.

Based on previous work that showed the amidated form of MrIA (**1**) to be the slightly more potent version of MrIA [7], this analog was used as the standard comparison compound for the NE uptake inhibition assay. A majority of MrIA analogues tested were significantly (>10-fold) less potent than the MrIA standard **1** (Figure 2). As shown in Figure 2, analogs **2**, **3**, **11**, **12-I**, and **17** maintained activity comparable to that of the standard 1. All these peptides contained the VCCGYKLCHXC sequence, with X = proline or hydroxyproline. These peptides do contain the disulfide scaffolding as MrIA and are N-terminal truncated with post-translational modifications, such as N-terminal amidation or proline oxidation. These modifications have in previous work been shown that they are well tolerated in regards to hNET activity [9]. A slight loss of activity was observed for peptides **12II** (non-native MrIA fold), and **13I** and **13II** (two isomers of the VCCGYKLCHOC sequence). None of the analogs significantly improved norepinephrine uptake inhibition.

Those analogs with comparable inhibitory activity to MrIA all maintained the three pharmacophore residues identified by Brust et al. [9], along with all four cysteine residues, while those with reduced activity lacked one or both of these pharmacophore characteristics. In peptides lacking all four cysteine residues, this was presumably due to conformational changes that did not allow the pharmacophore residues to be presented in a way that promoted optimal binding at the target. Interestingly, while the only peptide completely lacking all three pharmacophore residues (**10**) did have hNET inhibitory activity significantly less potent than the MrIA control, the activity was not significantly less than that of the other analogs that contained all three pharmacophore residues, but lacked one or more of the original cysteine residues. Therefore, while the pharmacophore residues are undoubtedly important in maintaining potent inhibitory hNET activity, they are not entirely necessary to elicit detectable hNET inhibition.

Of the peptide sequences that maintained both the pharmacophore residues and all four cysteine residues, two formed two different isomers upon oxidation (**12I** and **12II**, **13I** and **13II**). While each of these analogs retained activity greater than those with disrupted pharmacophores, for each set of isomers, one with the native 1-4, 2-3 disulfide fold was significantly more active than the other (**12I** > **12II**, **13II** > **13I**). Presumably, this was due to alternative disulfide connectivity that did not facilitate a hairpin gamma turn in the less active isomers.

Analog **18**, which contained a sulfonated tyrosine residue, also demonstrated reduced activity. This is congruent with previous work by Brust et al. [9], where it was also found that, with the exception of methylation, modification of the tyrosine residue greatly attenuated MrIA activity. The loss of activity may be due to sulfonation adding a negative charge to the previously uncharged residue, or because it adds bulk to a side chain that has a narrow spatial window for optimal binding [9], but is most likely some combination of the two. These results confirm the importance of the MrIA pharmacophore determined by Brust et al. [9] for the NET inhibition displayed by MrIA.

### 2.3. Calcium Influx Assays

**Figure 3:**
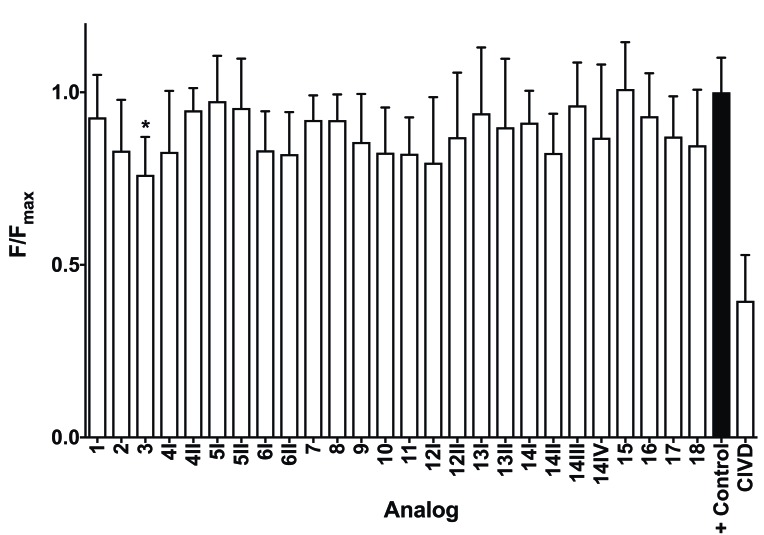
Average F/F_max_ values for MrIA analogs tested at 10 μM and CVID tested at 1 μM against Ca_V_2.2 with associated standard deviation bars. Analog **3** was found to have statistically significant antagonistic activity at a 99.9% confidence interval and is marked *.

When tested against the Ca_V_2.2 ion channel, the majority of analogs had no significant antagonistic effects (Figure 3). Only analog **3** significantly decreased activity at 10 μM with a 99.9% confidence interval, and the activity was not strong, as demonstrated by the comparison to the antagonistic activity of the ω-conotoxin CVID at 1 μM.

**Figure 4:**
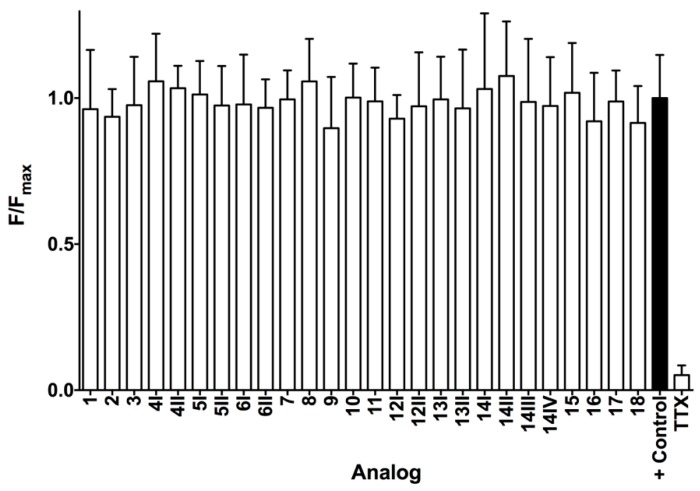
Average F/F_max_ values for MrIA analogs tested at 10 μM and TTX tested at 1 μM against voltage-gated sodium channels (Na_V_) with associated standard deviation bars.

MrIA analogs exhibited no significant antagonistic activity against the voltage-gated sodium channels expressed by SH-SY5Y cells at 10 μM concentrations (Figure 4). These include the Na_V_ subtypes 1.2, 1.3, 1.7 [10], and at lower levels, 1.4 and 1.5. Tetrodotoxin (TTX) at 1 μM concentration was used as an antagonist control for the Na_V_ channels. Furthermore, these MrIA analogs exhibited no significant agonistic activity against receptors that induce intracellular calcium responses in SHSY5Y [11] (data not shown).

## 3. Materials and Methods

### 3.1. Materials and Apparatus

Protected Fmoc-amino acid residues were purchased from Novabiochem or Auspep (Melbourne, AU) with the exception of the neopentyl-protected sulfotyrosine, which was purchased from Bachem. The Fmoc-Rink resin and Fmoc-Wang resin were purchased by Auspep and Peptides International (Louisville, USA), while the 2-chlorotrityl resin was purchased from Novabiochem. Peptide-synthesis grade dimethylformamide (DMF), dichloromethane (DCM), diisopropylethylamine (DIEA), and trifluoroacetic acid (TFA) were supplied by Auspep, while 2-(1H-benzotriazol-1-yl)-1,1,3,3- tetramethyluronium hexafluorophosphate (HBTU), triisopropylsilane (TIPS), HPLC-grade acetonitrile, ammonium bicarbonate, and ammonium acetate were supplied by Sigma-Aldrich (Sydney, AU). Assay reagents at the highest grade were also supplied by Sigma-Aldrich unless otherwise stated.

The Symphony automated peptide synthesizer was supplied by Protein Technologies Inc. Peptides were analysed using a Shimadzu Prominence HPLC system coupled to a PE Sciex API 150EX turbo ionspray mass spectrometer and purified on a Shimadzu Preparative HPLC system. The high-throughput FLIPR^Tetra^ fluorescent plate reader was supplied by Molecular Devices (CA, USA).

### 3.2. Peptide Synthesis

Peptides were synthesised either manually or on a Symphony (Protein Technologies Inc, AZ, USA) automated peptide synthesiser using standard Fmoc-SPPS methods [12]. C-terminal amidated peptides were assembled on Fmoc-Rink resins, while those with C-terminal carboxylation were assembled on preloaded Fmoc-Wang resins. The exception to this was the peptide with the sequence, ZGVCCGYKLCHP (**4I** and **4II**), which was assembled on a chlorotrityl resin to prevent diketopiperazine formation. Once assembled, peptides were cleaved with Trifluoroacetic acid (TFA)/water/Triisopropyl silane (TIPS) (90:5:5) for 3 hours, then were precipitated from chilled ether, filtered, and lyophilised from acetonitrile/water. Analytical RP-HPLC and electrospray mass spectrometry were used to confirm the purity and molecular masses of the final peptide products.

Reduced peptides were oxidised at pH 8.1 with a solution of 30% DMSO/70% NH_4_HCO_3_ for at least 2 hours at a concentration of 1 mg mL-1. The peptide with the sequence, LRNGVCCGYKLCHOC (**12I** and **12II**), was insoluble in NH_4_HCO_3_ and was therefore oxidised in a solution of 20% DMSO/80% TFA. Once oxidised, peptides were purified by RP-HPLC using a C18 column. The peptides were eluted using a linear gradient from 0%–40% solvent B (0.043% TFA in 90% acetonitrile) with solvent A being 0.05% TFA.

The sulfotyrosine analog **18** was synthesized in the same manner as the other analogs using comercial neopentyl-protected sulfotyrosine (Bachem SA, Bubendorf, CH). Peptide **18** was cleaved from the resin with TFA/water/TIPS (90:5:5) for 1.5 h at 0 °C. The oxidation of Peptide **18** was performed in 2 M ammonium acetate at 37 °C overnight and subsequently purified using RP-HPLC with a linear gradient between 0.1 M ammonium acetate and acetonitrile as recommended by Simpson et al. [13]. This method was employed to protect the highly acid labile sulfate group.

### 3.3. NE Uptake Inhibition Assay

The norepinephrine (NE) uptake assay was performed as previously described [14,15]. Consistant with these earlier studies, native MrIA inhibited hNET with a pIC_50_ of 5.87 ± 0.03. COS-7 cells (ATCC, VA, USA) were cultured in Dulbecco’s modified Eagle’s medium (DMEM) containing 10% fetal bovine serum (FBS) in 96-well plates. Cells were transiently transfected at the confluency of 90% with NET cDNA using 10 μL FuGENE (Roche, AU) and 2 μg DNA per 10^6^ cells. Twenty-four hours post-transfection, 10,000 cells from the overnight culture were evenly distributed per well in a 96-well plate format for an additional 24 hours incubation. An average cell count of approximately 50,000 cells per well was counted before uptake assays. NE uptake inhibition assay was performed using a fixed concentration of (^3^H)NE of 30 nM (40.5 Ci/mmol, Perkin Elmer, MA, USA) with increasing concentrations of peptide (**1**) and its peptide mimetics from 10^−4^ M to 10^−15^ M prepared in assay buffer (25 mM HEPES, pH 7.4, 125 mM NaCl, 1.2 mM MgSO_4_, 4.8 mM KCl, 1.2 mM KH_2_PO_4_, 1.3 mM CaCl_2_, 5.55 mM d-(+)-glucose, 1 mM ascorbic acid, 0.1% bovine serum albumin) using a 10-fold increment with a final volume of 50 μL per well in 96-well plates and incubated at 37 °C for 10 min, followed by two gentle washes using 100 μL assay buffer to remove excess (^3^H)NE. Cells were then lysed using 50 μL of 0.1 M NaOH with gentle shaking for 60 min. Lysed cells were transferred to the flexible 96-well plate (Perkin Elmer) with Optiphase Supermix scintillant (Perkin Elmer) for measurements. Each experiment was performed in triplicate and repeated three times.

### 3.4. Calcium Influx Assays

MrIA analogs (1 to 18, Table 1) were tested for antagonist activity at 10 μM concentrations against Ca_V_2.2 [16] and Na_V_ [10] ion channels using calcium influx assays and a high-throughput FLIPR^Tetra^ (Molecular Devices, CA, USA) fluorescent plate reader assay. SH-SY5Y cells were seeded in to black-walled, clear bottom 384-well imaging plates (Corning, NY, USA) at a concentration of approximately 40,000 cells per well two days prior to the assays. Cells were incubated in a Calcium 4 No Wash kit (Molecular Devices) in physiological salt solution (composition NaCl 140 mM, glucose 11.5 mM, KCl 5.9 mM, MgCl2 1.4 mM, NaH_2_PO_4_ 1.2 mM, NaHCO_3_ 5 mM, CaCl_2_ 1.8 mM, HEPES 10 mM) at 37 °C for 30 min. An addition of 10 μM nifedipine was made to the dye used to test for activity at Ca_V_2.2 in order to inhibit the L-type calcium channel activity. Using a cooled CCD camera at excitation 470–495 nM and emission 515–575 nM, 10 s baseline fluorescence readings were obtained, at which point the MrIA analogs were added to the cells and fluorescence readings were obtained every 2 s for 300 s. Standard agonists (90 mM KCl and 5 mM CaCl_2_ for Ca_V_2.2 and 45 μΜ veratridine for Na_V_s) were then added and further fluorescent readings were obtained every second for 300 s. Each assay was performed at least three times with three replicates of each analog per assay. Resulting maximum fluorescence readings were normalized to F/F_max_ using the average of the maximum fluorescence in positive control as F_max_. Analog activities were then compared to F_max_ using ordinary one-way ANOVA.

## 4. Conclusions

While some of the MrIA analogs synthesized maintained hNET inhibitory activity comparable to that of MrIA, none were able to significantly improve upon it. Indeed, MrIA may have arisen from such analogues and become stabilized in the genome optimized through positive selection. Those analogs lacking pharmacophore elements previously identified had greatly attenuated activity, supporting the MrIA pharmacophore identified by Brust et al. [9]. Calcium influx assays revealed that one analog produced off-target effects with antagonistic activity at Ca_V_2.2 channels. While this activity may not be biologically relevant, secondary conotoxin activities such as this have the potential to confer a competitive advantage. Based on this, we theorize that the “messiness” can provide the raw materials for evolutionary “experimentation” with the potential to enhance primary function and underpin the evolution of secondary function in conotoxins.

## Figures and Tables

**Table 1:** List of peptide analogs used in this study, including their peptide sequence, relative abundance in *Conus marmoreus* venom as compared to the amidated form of MrIA (from Duterte et al. [4]), and expected and observed masses. Cysteine residues are coloured red. “Z” denotes pyroglutamic acid, “O” denotes hydroxyproline, “*” denotes an amidated C-terminus, Y(SO_3_^−^) denotes sulfotyrosine.

Analog	Sequence	Relative Abundance	Expected MW (Da)	Observed MW (Da)
1	NGVCCGYKLCHPC *	100.00	1391.7	1392.0
2	NGVCCGYKLCHPC	49.77	1392.7	1392.8
3	ZGVCCGYKLCHPC	0.73	1389.7	1389.8
4I	ZGVCCGYKLCHP	0.05	1288.5	2577.6
4II				2577.6
5I	ZGVCCGYKLC	0.34	1054.3	2106.0
5II				2107.0
6I	NGVCCGYKLC	0.12	1057.3	2115.8
6II				2113.8
7	ZGVCCGYKL	0.03	951.1	951.6
8	ZGVCCGYKL *	0.01	950.1	950.4
9	NGVCCGYK	0.02	841.0	841.6
10	ILRGILRNGVCC *	0.02	1313.7	1314.0
11	GILRNGVCCGYKLCHPC	0.33	1831.2	1834.0
12-I	LRNGVCCGYKLCHOC	0.14	1678.0	1678.0
12-II				1677.8
13I	VCCGYKLCHOC	0.88	1237.5	1237.4
13II				1237.4
14I	CGYKLCHOC	0.21	1037.2	2073.6
14II				2072.8
14III				2073.0
14IV				2071.8
15	GYKLCHOC	0.04	934.1	934.6
16	YKLCHOC	0.02	877.1	877.6
17	GVCCGYKLCHPC	0.01	1278.6	1278.6
18	GVCCGY(SO_3_^−^)KLCHOC	0.11	1377.5	1376.6
